# Clean Spaces, Community Building, and Urban Stage: the Coproduction of Health and Parks in Low-Income Neighborhoods

**DOI:** 10.1007/s11524-022-00644-4

**Published:** 2022-06-14

**Authors:** Sanne Raap, Mare Knibbe, Klasien Horstman

**Affiliations:** grid.5012.6 0000 0001 0481 6099 Dep. Health, Ethics and Society, Health Inequities and Societal Participation (HISP), Care and Public Health Research Institute (CAPHRI), Faculty Health, Medicine, Life Sciences, Maastricht University, P.O. Box 616, 6200 MD Maastricht, The Netherlands

**Keywords:** Health disparities, Urban landscapes, Citizen interventions, Neighborhood parks

## Abstract

Green zones are potential contributors to health by mitigating disparities between low- and high-income neighborhoods. Against the background of different discourses about city parks—parks as restorative environments, parks as enabling places, and parks as sites for encounters between strangers—we ethnographically studied the coproduction of green spaces and health within low-income neighborhoods. We found three competing notions of urban green, each tied to different notions of neighborhood well-being. Parks as “clean spaces” create expectations of order and peace; parks as places of the community are related to play and activities; and parks as urban stage foster interactions between strangers. By generating experiences that encompass different conceptions of health, citizen-led events can contribute to a shift in the understanding of parks as sites of neighborhood decline to parks as places of hope and new beginnings.

## Introduction

The health benefits of green zones make urban greening an attractive strategy to address persistent health disparities between low- and middle- or high-income neighborhoods [1, 2]. Urban green spaces are associated with a reduction in air pollution, increased physical activity, mobility, relaxation, and restoration, amongst other benefits [3–6]. Currently, this health potential remains largely unfulfilled. In his review of 49 studies; Rigolon concluded that “low socioeconomic and ethnic minority people have access to fewer acres of parks, fewer acres of parks per person, and to parks with lower quality, maintenance, and safety than more privileged people” (p.160) [7].

Successfully addressing health disparities through urban greening requires more than the availability of green spaces as such [8–11]. Whereas improving physical park quality, adding facilities and organized programs can generate health benefits; enhanced understanding of the surrounding social environment is equally crucial as it directly pervades the experience of the physical environment [12–14]. For example, Seaman, Jones, and Ellaway stressed that the question of “walkability” includes perceptions of social cohesion at a community level. When local narratives determine who is a community member and thereby who can be considered a legitimate park user, inclusive measures and narratives can foster park use [15].

To strengthen the understanding of urban greening as a strategy to address health disparities, we did an ethnographic study in three low-income neighborhoods in Maastricht, the Netherlands. To capture the “mutually reinforcing and reciprocal relationship between people and place.” we adopted a relational view of parks that pays attention to the narratives about health and green spaces that coproduce local parks [16]. By focusing on residents’ practices and experiences of parks, we provide insights into the sociocultural understandings of city green and well-being as a pathway to understanding the health potentials of neighborhood parks [17].

In the following, we introduce three influential scientific discourses on health and parks. Next, we introduce the research setting and our ethnographic method. Thereafter, we present the research results; and finally, we discuss the implications of our findings for the health potentials of urban green spaces in low-income neighborhoods.

### Approaches to Urban Parks

Health scholarship that emerged in the 1980s and 1990s researched the link between health and green environments through the notion of restorative environments. Ulrich’s classical study demonstrates a significant decrease in recovery time from surgery for patients assigned a hospital room with a green view [18]. By elaborating on notions like “being away” and the relative absence of people, the literature on restorative environments suggests that we need restoration, as “the process of renewing (…) resources or capabilities diminished in ongoing efforts to meet adaptive demands” (p.273) to thrive within cities as environments of overstimulation [19–21]. The desire to escape the burdens of daily city life has been found to constitute an important reason for citizens’ visits to urban parks [22]. Parks can invoke experiences of order, for example through the managed beauty of flowerbeds resembling rural landscapes [23].

In a second approach, researchers have studied the interactions of individuals with their environments [16, 24]. An interactive approach to city–nature finds expression in the notion of urban parks as “enabling environments” or “enabling places” [24, 25]. The literature on enabling places theorizes health as a form of agency and personal development. In this view, health is not derived from “exposure” but is mediated by social, affective, and material relations. For example, the aesthetic experience of gardening, through getting one’s hands dirty and growing food, fosters both an emotional connection with the garden and supportive relationships with others [26]. Community gardens, as sites of learning through play and gardening, as well as small societal experiments in local governance, form instructive examples of the enablement approach [27].

Restorative and enablement approaches theorize the health benefits of parks as offering what much of daily city life supposedly lacks: beauty and quiet or community, respectively. A third, urbanist approach, on the other hand, treats parks as an integral part of the wider urban landscape. The quality of a park is determined not solely by its design or perceived naturalness but by its integration with surrounding amenities [28]. Like squares and streets, parks form an important part of the city’s public realm, which offers a setting for genuine and profound interaction [29]. In line with the urbanist idea of “the city as a stage” or “theater of social action,” recent studies have revealed parks to be popular sites for public realm interactions like play, public sociability, and people watching [30–32]. To public realm theorists, parks can provide a salutary and open environment for interactions between strangers and thereby foster experiences of equality and tolerance [33].

## Research Setting and Method

### The Neighborhoods

Our research setting comprised three postwar neighborhoods built around 1960 in Maastricht, the Netherlands. The neighborhoods are characterized by their parochial design, i.e., church and shops in the center. However, over the years, many public facilities have disappeared. Green spaces constitute another main characteristic of the neighborhoods, which encompasses two designated parks, a few off-leash dog areas, and many so-called “green floors” and “green wedges”.

The three neighborhoods are quite diverse; more than 40% of the residents are migrants, of whom 23% have a non-Western background. All three neighborhoods are currently regarded as low-income and local public health statistics show a high burden of disease. Around 40% of all neighborhood residents have a chronic disease compared to the city average of 26%. Unemployment, loneliness, and disability percentages reveal a similar pattern [34].

### Actions: University with the Neighborhood and Neighborhood Campsite

Since 2017, neighborhood residents and researchers from the University of Maastricht work together as “University with the Neighborhood” to facilitate an exchange of ideas about neighborhood well-being. In 2018, the working group Social Green, consisting of citizens and researchers, was founded to transform asocial green spaces into social green zones, with an initial focus on a neighborhood park. To transform this park, the working group engaged in dialogue with other residents and experimented with potential uses of the park in 2019, like a performance by a local folk singer, a neighborhood talk show, an informal pop-up tea garden, an outdoor philosophy café, campfires, and dancing in the park. In cooperation with the working group, an additional group of volunteers and social workers organized a neighborhood campsite to bring people from different life worlds together. Inhabitants of the neighborhoods bordering the park were invited to camp in the park for two nights and participate in numerous activities, such as a barbecue, a campfire, theater, and tai chi lessons. Residents of the three studied neighborhoods were informed about the events by means of flyers, social media, and local news media. Working group activities were free of charge; participation in the campsite event required a fee of 15 euros, with a 50% discount for those on social benefits.

### Method

This article draws on 3 years of ethnographic fieldwork between 2017 and the beginning of 2020. The first author conducted 15 walking interviews [35] with neighborhood residents between 2017 and 2018, which enabled her to connect neighborhood experiences and memories with specific places, including green zones. Second, the first author conducted participant observations during working group activities in the park, including preparations and evaluation meetings. Third, numerous informal conversations and observations in the park and at neighborhood meetings were undertaken to generate data on local understandings of green zones. Finally, students administered 40 questionnaires, containing open questions about residents’ experiences with neighborhood green spaces. Relevant local media products and local policy documents were analyzed to contextualize the findings.

We acquired consent for all interviews and audio-recorded evaluation meetings. Additionally, all participants were informed about the nature and aims of the research throughout the research process. This disclosure abided by established codes of conduct in anthropology, which views informed consent as an ongoing process [36]. Detailed field notes and transcribed audio recordings were analyzed supported by the software program QRS NVivo11. Through an iterative process of deductive and inductive coding, guided by restoration, enablement, and (public) sociability as sensitizing concepts, we distilled three main themes in relation to neighborhood green spaces: green spaces as clean spaces, as sites for community wellbeing, and green spaces as sites for stranger interaction [37].

## Results

The analysis showed that the meaning of neighborhood green spaces is contested. Against broader narratives of neighborhood decline, green spaces were constructed as unclean and uncared for, as (not) enabling community interactions, and as places that open up new horizons of neighborhood life.

### Green Spaces as Clean Spaces

Living in a green neighborhood can be a salutary privilege, especially in the summer season.

*“If, in the summer, you come back from work, or you enter the neighborhood – yes, that is a breath of fresh air.”* (walking interview)

*“On that account, living in the neighborhood is good (…); so much green, they hardly have that anywhere (...). Yes, I am very happy with that.”* (walking interview)

While residents regarded their green neighborhood as a source of pride, they also expected green spaces to be neat and well maintained. Unfortunately, the daily reality rarely lived up to this expectation. Especially the decay of a local pond turned into a swamp, and a rose garden, now in ruins, were experienced as a loss.

*“It is a shame that it [the park] is neglected. At least when I moved here, it was really nice, with roses. It was called a rosarium (...).But well; they apparently stopped spending money on that.”* (walking interview)

Dumped rubbish, discarded needles, dog feces, and broken benches do not only impede mobility and spoil the beauty of neighborhood green spaces, they were highlighted as representing residents’ concerns about the state of their neighborhood and emblematic of its disappearance from the municipality’s radar. While showing the interviewer a site that was overgrown with weeds and littered with derelict benches and rubbish, one resident described his experience as follows:

*“These are hidden neighborhoods actually. But we [do] live here.”* (walking interview).

On the other hand, some residents experienced such “hiddenness” as a blessing. One interviewee in particular stressed how he found peace in the poorly maintained park, as it kept the crowds away. Another resident, who walked the park regularly, described it as one of the few places where local alcoholics, who were not hurting anyone, were tolerated.

The experience of neglect was not only manifested in relation to a lack of maintenance per se but also stemmed from the municipality’s recent ecological approach to city–nature, which does not support clean green environments:

*“The green (...) used to be sprayed. And then it was clean;* [however], *they are not allowed to do that anymore.”* (walking interview)

Ecological policies, like not mowing the grass, are controversial. Some parents do not let their children play on high grass.

*“Many (...) see it as yes, ticks and this and that, and people are afraid of ticks at the moment. We always played in the grass in my youth (...), but this is an eye sore to many residents; they do not understand what the idea is, or they are scared.”* (walking interview)

Thus, some residents pointed out that by relinquishing the means to control city–nature, nature intrudes private realms and raises health concerns.

Residents who understood the connection between parks and well-being as a matter of cleanliness demanded that city green spaces constituted a well-controlled backdrop, nonintrusive and risk free. “Clean” parks however may clash with a wish for naturalness. Some residents contested the clean look of a newly developed park with large lawns, asphalt walking paths, few trees, and no bushes:

*“Please do you call this a park, a waste of land!!! Gravel and asphalt everywhere (…) a park is supposed to be beautiful with trees, lots of plants maybe a pond or something like that. A park is nature!! This is just UGLY!!! A disgrace for the neighborhood I live in.”* (post on the public municipal Facebook page)

Members of the Social Green working group also considered a “clean” park to be uninviting and met with municipal landscape architects to discuss the introduction of more natural diversity. A few months later, 46 new trees were planted, and a citizen initiative was started to monitor the butterfly population in the wilder flower field, slowly changing the park’s appearance.

### Grass Does Not Grow Communities

Interviewees also emphasized the social life of green zones, often by talking about children’s play. While passing a green patch of grass surrounded by row houses, one resident described how it was once a real hotspot for children:

*“(...) as soon as you would leave school (...) it was like, at that and that time, we are at the field (…) than we played rounders, and you received extra points when you hit the high-rise building.”* (walking interview)

Parks were also remembered as a social place for adults. While passing an empty park, one resident shared a memory of the football club that used to be located there. Half of the neighborhood once gathered here on Saturdays:

*“Look, it used to be like this – say, the football club, that was a football club – [but] it was actually a place where people would meet each other (...). There was a wooden shed, and over there was a canteen. You could play table football, and those people [older men] came there to play cards.”* (walking interview)

Residents’ descriptions of spontaneous and playful interactions indicate that, in the past, green spaces were social spaces. However, sports clubs moved outside of the neighborhoods, small shops closed down, and many children now attend schools elsewhere. Currently, without supportive social infrastructures, empty stretches of grass offer residents few opportunities to watch, meet, and interact with others. Dogs and their owners represent a notable exception. Dogs give people a reason to leave their houses and create the rare occasion to engage in a conversation:

*“Well [usually] they [other residents] just pass (...), but when someone passes who also owns a dog, then it is better, then you have something to say (...). Yes, a pet is actually important.”* (walking interview)

Dog owners especially make new acquaintances in off-leash areas. Not unlike other park users; however, they are often dissatisfied with the quality of off-leash areas, for which the municipality has declined requests for streetlights and benches. Moreover, unlike the football club in earlier days, the loose community of dog owners does not attract other park users.

Can current neighborhood parks contribute to building a new neighborhood community? The municipality thinks that this is the case and considers citizen participation an important means for park improvement.

*“It is possible to manage a piece of public green, with the aim to bring the green to a higher level (...). It is best when you start working on public green together with neighbors, or even the whole street. This will make the environment belong more to you together.”* (website municipality)

This message, however, does not resonate with those residents who, after witnessing the dumping of rubbish and vandalism for years, feel that cleaning up green spaces is the municipality’s responsibility. Some have given up on the park’s potential as a social space altogether:

“[F]or my part, they asphalt it [the park] completely.” (field notes)

Moreover, some residents noted that citizen initiatives are often restricted by municipal policies. Difficulties surrounding a New Year’s Eve neighborhood tradition close to a green wedge provide an example of municipal policies that ultimately make the neighborhood belong less to residents:

*“People miss the sociability (...) the municipality is a little hypocritical, saying you can’t do Christmas tree burning anymore, it is too close to the neighborhood. Well look at the open spaces there [pointing to the wedge] (…). Yes, I think the municipality wants citizens to do things themselves, but (…) they crush initiatives.”* (walking interview)

Grass, in other words, does not grow communities. Green spaces need to function as social infrastructures conducive to communities gathering for play and relaxation. Like the football club in its heyday, campfires can attract people, almost without effort. Realizing this, in 2019, citizens and researchers organized temporary transformations of one of the neighborhood parks.

### Sublimating a Low-Income Neighborhood

Activities organized by citizens in one neighborhood park in 2019 aimed to transform the park into a social destination, which eventually led neighborhood residents to see the neighborhood in a new light. First, the citizen-led activities transformed the park from a display of decline into a social space amenable to rearrangement and reinvention. The campsite, for a weekend, turned the park into a completely new place:

*“The park was emptied of dog faeces and other rubbish by volunteers and a steel construction was transformed into a reception desk to welcome guests’. Larger and smaller tents occupied the grass, and the in-between space was used for activities like yoga and puppeteer performances. A large tree turned into a stage for circus acts, and free second-hand gifts were hung in a “gift” tree. The off-leash area became a sheepfold, now forbidden for dogs but open for children who wanted to cuddle the sheep.”* (field notes)

The summer events that followed the campsite, similarly created new configurations of people by temporarily redesigning the park. For example, a pop-up tea garden succeeded “to make encounters happen” by attracting older people looking for an opportunity to chat.

Some elements that remained after the activities had ended continued to bring small changes to the park, such as the installation of a few tree trunks for people to sit on:

*“Those tree trunks [in the park] – that is a success (...). They [children] are still dragging them around (…).”* (residents’ conversation, evaluation meeting)

Creating a new setting did not mean that vandalism was no longer a risk. Willow huts were destroyed not long after they had been built. As building as well as playing in them had brought people together, the working group, however, concluded that “it has done its job.”

The citizen-led events also generated new or forgotten forms of sociability. Although initially, many residents greeted the announcement of the campsite with some skepticism, after witnessing its construction, many offered to help, for example by bringing wood for the campfire. As a campsite participant described her experience:

“Together with the people, it was simply an experience of togetherness.” (participant).

The events succeeded, as a resident put, in “getting people out of their houses”—no small achievement in their neighborhood. Seeing how one could participate stimulated others to do so.

Finally, the park events not only permitted a temporary escape from daily life but also supported residents to imagine new beginnings. The campsite, for example, included a fortune teller, and, on a decorated altar in a circus tent, the opportunity was given for participants to marry for a day. One couple used this opportunity to spiritually reconfirm their marriage, as the husband had just returned from rehab. The events illuminated possible new futures for neighborhood life as well. Residents were proud that “finally, something had happened” in their neighborhoods, which brought together different ethnicities, young and old, healthy and unhealthy. Participants described the campsite as a sublime experience: an achievement that was “just like a movie,” “a culture trip,” and in which “everything happened naturally.” They had become part of a new, uplifting sort of neighborhood, one positively portrayed by local media, endorsed by municipal officials, and “discovered” by people from outside of the neighborhoods. Most importantly, participants noted that they now knew and greeted more neighbors, making the events and their outcomes palpable even after the last elements had been removed from the park.

## Discussion and Conclusion

Our analysis of residents’ experiences of green spaces in three low-income neighborhoods highlights three different coproductions of health and parks. First, residents expected the park to be “clean spaces.” From the perspective of restorative environments, “clean spaces” express a desire for order. However, rather than forming an escape from neighborhood nuisances, poorly maintained green spaces served as a constant reminder of the neighborhood’s problems [22]. Second, residents understood parks as sites for play and community maintenance. Comparing residents’ perspectives to the enablement approach to parks, which understands health as a matter of agency; it becomes evident that empty stretches of grass devoid of (social) infrastructures do not generate interactions [24]. Third, our analysis of the citizen-led events highlights how several rearrangements transformed a neighborhood park, literally (e.g., dance floor, performances) and metaphorically, into a theater for people watching and spontaneous encounters.

By highlighting how different conceptions of green spaces intersect, our study contributes to a multilayered understanding of the health potential of urban greening. First, our analysis contributes to research examining the health-promoting character of green spaces as shaped by social environments and narratives [14, 38, 39]. We found that competing narratives exist with respect to ecology, order, community, and public sociability but that an overarching narrative of neighborhood neglect saturated most inhabitants’ perspectives. This is an important finding to consider, as a lack of insight or recognition by designers and policymakers into the local narratives that coproduce healthy parks—by, for example, overlooking experiences of neglect—could reinforce spatial stigma [40].

Secondly, by drawing on more than one academic tradition, i.e., psychology, anthropology, and urban sociology, our study aligns with studies that understand health as multiple and embedded in local practices [41–43]. This constitutes a challenge for park design and intervention. Turning parks into sanitized spaces is likely to generate peace of mind for some but can be detrimental to the well-being of others with regard to social encounters and liveliness.

Finally, our research underscores that the health benefits of urban green spaces in low-income neighborhoods can only be realized when struggles over the social meanings of green spaces are taken seriously. Building on literature on the role of parks in stimulating contact between different (ethnic) groups, we found that although multilayered park narratives can be competitive, they are neither immutable nor mutually exclusive [31, 32, 44]. By fostering palpable experiences of being away, and by enabling interactions between strangers, citizen park interventions such as those described in our research can contribute to the transformation of low-income neighborhoods from places of decay to spaces of hope. The rearrangement of parks as arenas for new beginnings offers a unique pathway toward a positive neighborhood identity and the well-being of residents.
